# Nicotine impact on melanogenesis and antioxidant defense system in HEMn-DP melanocytes

**DOI:** 10.1007/s11010-014-2116-1

**Published:** 2014-06-19

**Authors:** Marcin Delijewski, Dorota Wrześniok, Michał Otręba, Artur Beberok, Jakub Rok, Ewa Buszman

**Affiliations:** Department of Pharmaceutical Chemistry, Faculty of Pharmacy, Medical University of Silesia, Jagiellońska 4, 41-200 Sosnowiec, Poland

**Keywords:** Nicotine, Melanocytes, Melanogenesis, Tyrosinase, Antioxidant enzymes

## Abstract

Nicotine is a compound of tobacco plants and is responsible for addictive properties of tobacco which is used by about one billion of smokers all over the world. Recently, nicotine has drawn even more attention due to its presumed neuroprotective and antioxidant features as far as common use in various forms of smoking cessation therapies. It is suggested that nicotine may be accumulated in human tissues containing melanin. This may in turn influence biochemical processes in human cells producing melanin. The aim of this study was to examine the impact of nicotine on melanogenesis and antioxidant defense system in cultured normal human melanocytes (HEMn-DP). Nicotine induced concentration-dependent loss in melanocytes viability. The value of EC_50_ was determined to be 2.52 mM. Nicotine modulated melanin biosynthesis in normal human melanocytes. Significant changes in hydrogen peroxide content and cellular antioxidant enzymes: SOD, CAT, and GPx activities were stated in melanocytes exposed to nicotine, which indicates alterations of antioxidant defense system. The results obtained in vitro may explain a potential influence of nicotine on biochemical processes in melanocytes in vivo during long-term exposition to nicotine.

## Introduction

Nicotine being a main compound of tobacco plants (*Nicotiana*) is responsible for tobacco addiction that leads to high occurrence of smoking-related premature deaths reaching even a number of 435,000 diseases each year in the United States, mainly due to cancer, cardiovascular and pulmonary diseases induced by toxins in tobacco smoke [1]. On the other hand, nicotine is a commonly used agent for smoking cessation therapies and promising substance in pharmacological attempts because of its presumed neuroprotective and antioxidant properties [2–4].

Nicotine can be absorbed from cigarette smoke through lungs, from chewing tobacco through oral mucosa, or from nicotine replacement therapy (NRT) that consists of nicotine gums, inhalers, nasal sprays, sublingual tablets, lozenges, and from transdermal patches through skin. After reaching brain, it causes a reinforcement effect, which rapidity depends on speed, quantity, and form of absorption. The fastest and the most effective delivery of nicotine is the tobacco smoke, as it gives a potentiality of adjusting the individual level of nicotine that reaches brain, by maintaining speed, frequency, and depth of puffs by each cigarette. Differently from tobacco smoke, that delivers nicotine directly to the lungs, bioavailability of nicotine through mucosa of the oral cavity is about 50–80 %. In case of transdermal delivery, nicotine appears in bloodstream after the initial lag time of about 1 h [5, 6]. Tobacco smoke is thereby the most effective way of nicotine absorption which is, however, abundant in accompanied cancerogenic compounds. Taking into account the effectiveness and safety of NRT, one can consider the contribution of melanin in the distribution of nicotine in body tissues.

Melanin is a natural polymeric pigment produced in the interior of melanocytes, in specialized membrane-bound organelles, melanosomes. It occurs in the skin, as well as in other organs and structures such as the eye, hair, inner ear, heart, lungs, liver, lymphocytes, and brain. Melanin is synthesized in multistep process called menalogenesis in which three main melanogenic metalloenzymes are involved: tyrosinase, tyrosinase-related protein 1 (TRP1), and tyrosinase-related protein 2 (TRP2). The most critical enzyme for the whole process is tyrosinase [7, 8]. Melanin is traditionally believed to work as UV radiation absorbent as well as antioxidant agent and free radicals scavenger [9]. Being a diversified polymer containing various reactive centres, melanin is capable of binding a number of chemicals and medicinal substances, including aminoglycoside antibiotics [10], fluoroquinolones [11], anticancer agents [12], and psychotropic drugs [13].

The literature suggests that nicotine may be accumulated in human tissues containing melanin and retention in these tissues may increase melanin synthesis. This may in turn influence nicotine metabolism, level of nicotine addiction, and good results of smoking cessation therapies [14]. Considering this fact it can be assumed that the presence of nicotine could lead to melanin pigmentations, which were observed in oral mucosa of smokers and even children exposed to second hand cigarette smoke [15–17].

The major findings from previous research on nicotine’s affinity to melanin include proofs for formation of nicotine adducts to melanin’s intermediate, dopaquinone, during in vitro synthesis of melanin in presence of nicotine [18], tight association of nicotine to melanin [19, 20], high incorporation of nicotine to the pigmented hair during hair formation [21], and accumulation and long-term retention of nicotine in melanin-containing tissues in mice [22]. However, until now there were no exact studies on the effect of nicotine on human cells, focusing on biochemical processes involving biosynthesis of melanin and antioxidant status in cell culture. Interactions between nicotine and melanin may be of particular importance in case of dark-pigmented individuals that have more melanin and may sequester nicotine in the body. This accumulation of nicotine may be associated with reduction of nicotine clearance, and increase in time one is exposed to nicotine and higher dependence that may result in lower tobacco cessation rates [23].

Previously, we documented that fluoroquinolones: ciprofloxacin [11] and lomefloxacin [24], as well as aminoglycoside antibiotics: amikacin [25], kanamycin [26], netilmicin [27], and streptomycin [28] suppressed melanin biosynthesis in human light pigmented melanocytes. Our studies also demonstrated that the analyzed drugs affected antioxidant enzymes activities in HEMa-LP melanocytes.

In order to estimate the nicotine-melanin relationship in living cells, we examined the effect of nicotine on viability, melanogenesis and antioxidant status in cultured normal human melanocytes (HEMn-DP).

## Materials and methods

Nicotine, phosphated-buffered saline (PBS), 3,4-dihydroxy-l-phenylalanine (l-DOPA), and amphotericin B were purchased from Sigma-Aldrich Inc. (USA). Neomycin sulfate was obtained from Amara (Poland). Penicillin was acquired from Polfa Tarchomin (Poland). Growth medium M-254 and human melanocyte growth supplement-2 (HMGS-2) were obtained from Cascade Biologics (UK). Trypsin/EDTA was obtained from Cytogen (Poland). Cell Proliferation Reagent WST-1 was purchased from Roche GmbH (Germany). The remaining chemicals were produced by POCH S.A. (Poland).

### Cell culture

The normal human epidermal melanocytes (HEMn-DP, Cascade Biologics) were grown according to the manufacturer’s instruction. The cells were cultured in M-254 basal medium supplemented with HMGS-2, penicillin (100 U/ml), neomycin (10 μg/ml), and amphotericin B (0.25 μg/ml) at 37 °C in 5 % CO_2_. All experiments were performed using cells in the passages 5–8.

### Cell viability assay

The viability of melanocytes was evaluated by the WST-1 (4-[3-(4-iodophenyl)-2-(4-nitrophenyl)-2H-5-tetrazolio]-1,3-benzene disulphonate) colorimetric assay. WST-1 is a water-soluble tetrazolium salt, the rate of WST-1 cleavage by mitochondrial dehydrogenases correlates with the number of viable cells. In brief, 5,000 cells per well were placed in a 96-well microplate in a supplemented M-254 growth medium and incubated at 37 °C and 5 % CO_2_ for 48 h. Then, the medium was removed and cells were treated with nicotine solutions in a concentration range from 0.0001 to 10 mM. After 21-h incubation, 10 μl of WST-1 was added to 100 μl of culture medium in each well, and the incubation was continued for three hours. The absorbance of the samples was measured at 440 nm with a reference wavelength of 650 nm, against the controls (the same cells but not treated with nicotine) using a microplate reader UVM 340 (Biogenet, Poland). The controls were normalized to 100 % for each assay and treatments were expressed as the percentage of the controls.

### Measurement of melanin content

The melanocytes were seeded in T-25 flasks at a density of 1 × 10^5^ cells per flask. Nicotine treatment in a concentration range from 0.0001 to 1.0 mM began 48 h after seeding. After 24 h of incubation, the cells were detached with trypsin/EDTA. Cell pellets were placed into Eppendorf tubes, dissolved in 100 μl of 1 M NaOH at 80 °C for 1 h, and then centrifuged for 20 min at 16,000*g*. The supernatants were placed into a 96-well microplate and absorbance was measured at 405 nm—a wavelength at which melanin absorbs light [29]. Melanin content in nicotine-treated cells was expressed as the percentage of the controls (untreated melanocytes).

### Tyrosinase activity assay

Tyrosinase activity in HEMn-DP cells was determined by measuring the rate of oxidation of l-DOPA to DOPAchrome, according to the method described by Kim et al. [30] and Busca et al. [31], with a slight modification. The cells were cultured at a density of 1 × 10^5^ cells in T-25 flasks for 48 h. After 24-h incubation with nicotine (concentration range from 0.0001 to 1.0 mM), cells were lysed and clarified by centrifugation at 10,000*g* for 5 min. A tyrosinase substrate l-DOPA (2 mg/ml) was prepared in the same lysis phosphate buffer. 100 μl of each lysate was put in a 96-well plate, and the enzymatic assay was initiated by the addition of 40 μl of l-DOPA solution at 37 °C. Absorbance was measured every 10 min for at least 1.5 h at 475 nm using a microplate reader. Tyrosinase activity was expressed as the percentage of the controls (untreated melanocytes).

### Superoxide dismutase (SOD) assay

Superoxide dismutase (SOD) activity was measured using an assay kit (Cayman, MI, USA) according to the manufacturer’s instruction. This kit utilizes a tetrazolium salt for the detection of superoxide radicals generated by xanthine oxidase and hypoxanthine. One unit of SOD was defined as the amount of enzyme needed to produce 50 % dismutation of superoxide radical. SOD activity was expressed in U/mg protein. The samples were taken from the same lysates that were used for tyrosinase activity assay.

### Catalase (CAT) assay

Catalase (CAT) activity was measured using an assay kit (Cayman, MI, USA) according to the manufacturer’s instruction. This kit utilizes the peroxidatic function of CAT for determination of enzyme activity. The method is based on the reaction of the enzyme with methanol in the presence of an optimal concentration of H_2_O_2_. The formaldehyde produced is measured colorimetrically with 4-amino-3-hydrazino-5-mercapto-1,2,4-triazole (Purpald) as the chromogen. One unit of CAT was defined as the amount of enzyme that causes the formation of 1.0 nmol of formaldehyde per minute at 25 °C. CAT activity was expressed in nmol/min/mg protein. The samples were taken from the same lysates that were used for tyrosinase activity assay.

### Glutathione peroxidase (GPx) assay

Glutathione peroxidase (GPx) activity was measured using an assay kit (Cayman, MI, USA) according to the manufacturer’s instruction. The measurement of GPx activity is based on the principle of a coupled reaction with glutathione reductase (GR). The oxidized glutathione (GSSG) formed after reduction of hydroperoxide by GPx is recycled to its reduced state by GR in the presence of NADPH. The oxidation of NADPH is accompanied by a decrease in absorbance at 340 nm. One unit of GPx was defined as the amount of enzyme that catalyzes the oxidation of 1 nmol of NADPH per minute at 25 °C. GPx activity was expressed in nmol/min/mg protein. The samples were taken from the same lysates that were used for tyrosinase activity assay.

### Hydrogen peroxide (H_2_O_2_) assay

Hydrogen peroxide (H_2_O_2_) content was measured using an assay kit (Cell Biolabs, Inc., USA) according to the manufacturer’s instruction. This method is based on the ability of sorbitol to convert peroxide to a peroxyl radical, which oxidizes Fe^2+^ into Fe^3+^. Then Fe^3+^ reacts with an equimolar amount of xylenol orange in the presence of acid to create a purple product that absorbs light at maximal wavelength 595 nm. The antioxidant—butylated hydroxytoluene (BHT) is provided to prevent further undesirable chain peroxidation. Hydrogen peroxide content in the samples was expressed in µmol/mg protein. The samples were taken from the same lysates that were used for tyrosinase activity assay.

### Statistical analysis

In all experiments, mean values of at least three separate experiments (n = 3) performed in triplicate ± standard error of the mean (S.E.M.) were calculated. The results were analyzed statistically using GraphPad Prism 6.01 Software. A value of *p* < 0.05 (*) or *p* < 0.005 (**), obtained with a Student’s *t* test by comparing the data with those for control (cells without nicotine), was considered statistically significant.

## Results

### The effect of nicotine on cell viability

**Fig. 1:**
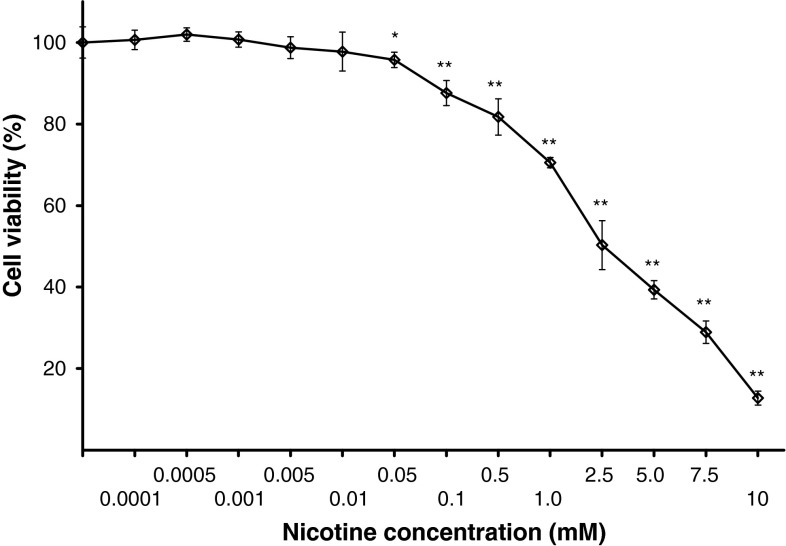
The effect of nicotine on viability of melanocytes. Cells were treated with various nicotine concentrations (0.0001–10 mM) and examined by the WST-1 assay. Data are expressed as % of cell viability. Mean values ± S.E.M. from three independent experiments (*n* = 3) performed in triplicate are presented. *p < 0.05 versus the control samples; **p < 0.005 versus the control samples

Melanocytes were treated with nicotine in a range of concentrations from 0.0001 to 10 mM for 24 h (Fig. 1). The cell viability was determined by the WST-1 test assay. At a relative low nicotine concentrations (0.0001–0.01 mM), the loss in cell viability was not statistically significant. Treatment of cells with 0.05, 0.1, 0.5, 1.0, 2.5, 5.0, 7.5, and 10 mM of nicotine led to the loss of about 4.2, 12.4, 18.3, 29.5, 49.7, 60.7, 71.1, and 87.2 % in the cell viability, respectively. The value of EC_50_ (i.e., the concentration of a drug that produces loss in cell viability by 50 %) was determined to be 2.52 mM.

### The effect of nicotine on melanization process

**Fig. 2:**
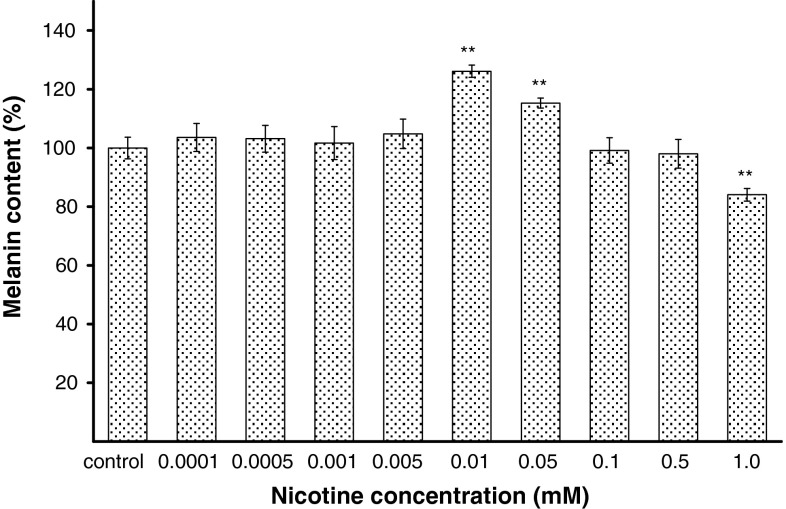
The effect of nicotine on melanin content in melanocytes. Cells were treated with various nicotine concentrations (0.0001–1.0 mM) for 24 h, and melanin content was measured as described in “Materials and methods” section. Results are expressed as percentages of the controls. Data are mean ± SEM of at least three independent experiments (*n* = 3) performed in triplicate. **p < 0.005 versus the control samples

The effectiveness of melanization process was estimated by measuring the melanin content and cellular tyrosinase activity in melanocytes treated with nicotine in a range of concentrations from 0.0001 to 1.0 mM for 24 h. Nicotine in concentrations from 0.0001 to 0.005 mM and in concentrations of 0.1 and 0.5 mM had no effect on melanin content (Fig. 2). In cells treated with nicotine at concentrations of 0.01 and 0.05 mM for 24 h, melanin production increased by about 26 and 15 %, respectively. Treatment of cells with 1.0 mM of nicotine reduced melanin content by about 16 %.

**Fig. 3:**
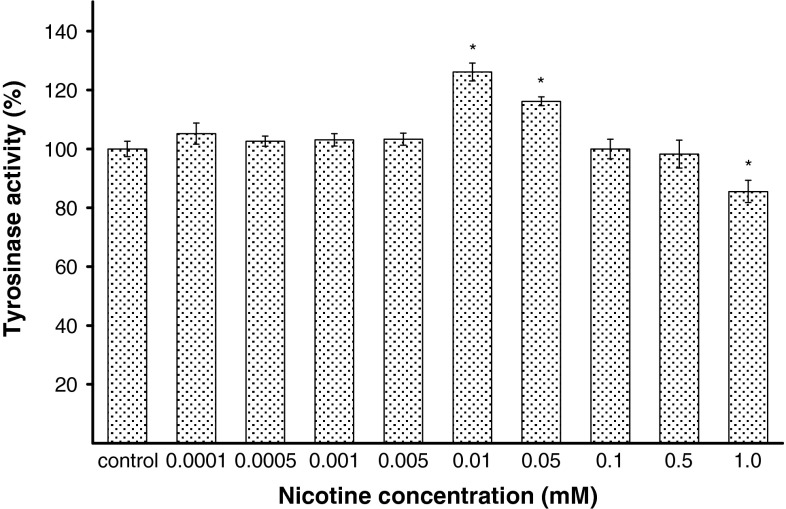
The effect of nicotine on tyrosinase activity in melanocytes. Cells were treated with various nicotine concentrations (0.0001–1.0 mM) for 24 h, and tyrosinase activity was measured as described in “Materials and methods” section. Data are mean ± SEM of at least three independent experiments (*n* = 3) performed in triplicate. *p < 0.05 versus the control samples

Tyrosinase activity in HEMn-DP cells treated with nicotine also changed in a manner correlating well with the effect on melanin formation (Fig. 3). After 24-h incubation with nicotine, tyrosinase activity was suppressed to 86 % at 1.0 mM when compared with the controls. In cells treated with nicotine at concentrations of 0.01 and 0.05 mM for 24 h, tyrosinase activity increased by about 26 and 16 %, respectively. Nicotine in the range of concentrations from 0.0001 to 0.005 mM and in concentrations of 0.1 and 0.5 mM had no effect on the cellular tyrosinase activity.

### The effect of nicotine on antioxidant enzymes activities

**Fig. 4:**
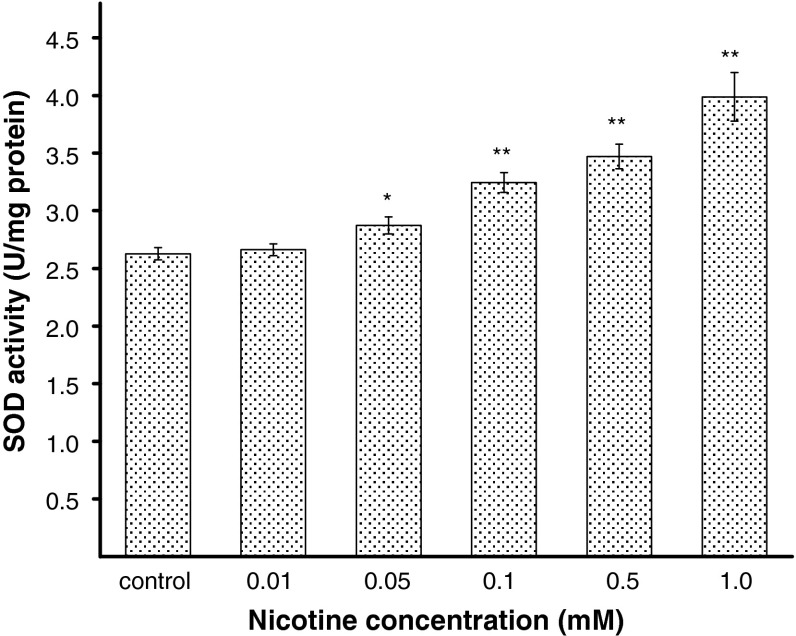
The SOD activity in HEMn-DP cell after 24 h incubation with 0.01, 0.05, 0.1, 0.5, or 1.0 mM of nicotine. Data are mean ± SEM of at least three independent experiments (*n* = 3) performed in triplicate. *p < 0.05 versus the control samples; **p < 0.005 versus the control samples

**Fig. 5:**
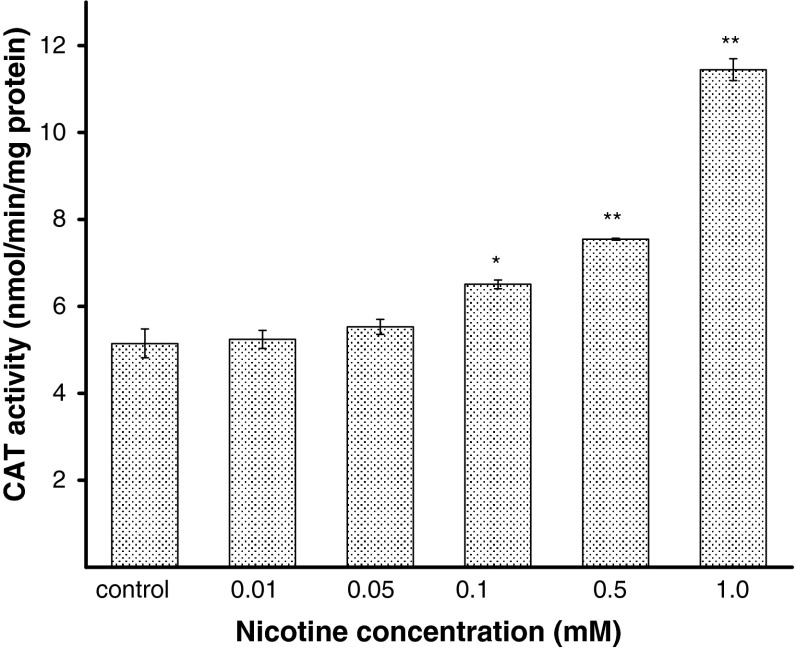
The CAT activity in HEMn-DP cell after 24 h incubation with 0.01, 0.05, 0.1, 0.5, or 1.0 mM of nicotine. Data are mean ± SEM of at least three independent experiments (*n* = 3) performed in triplicate. *p < 0.05 versus the control samples; **p < 0.005 versus the control samples

**Fig. 6:**
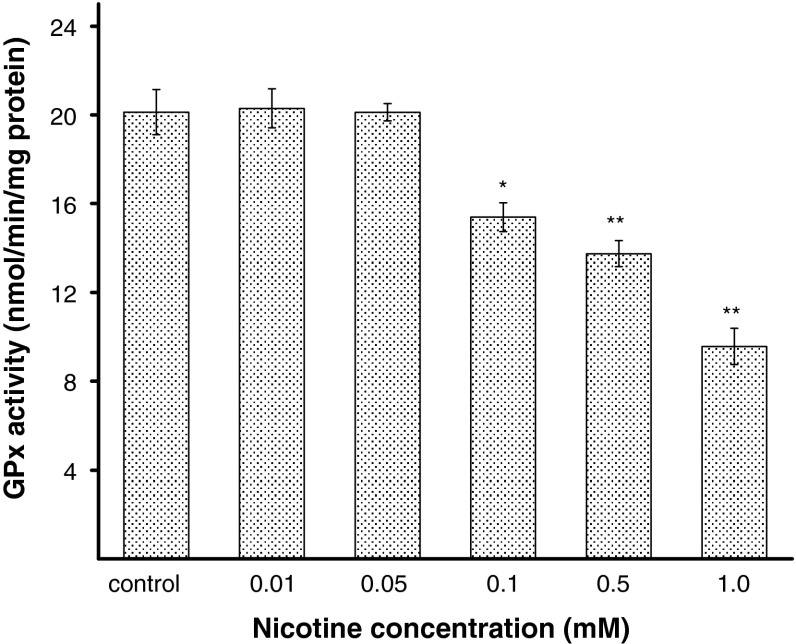
The GPx activity in HEMn-DP cell after 24 h incubation with 0.01, 0.05, 0.1, 0.5, or 1.0 mM of nicotine. Data are mean ± SEM of at least three independent experiments (*n* = 3) performed in triplicate. *p < 0.05 versus the control samples; **p < 0.005 versus the control samples

**Fig. 7:**
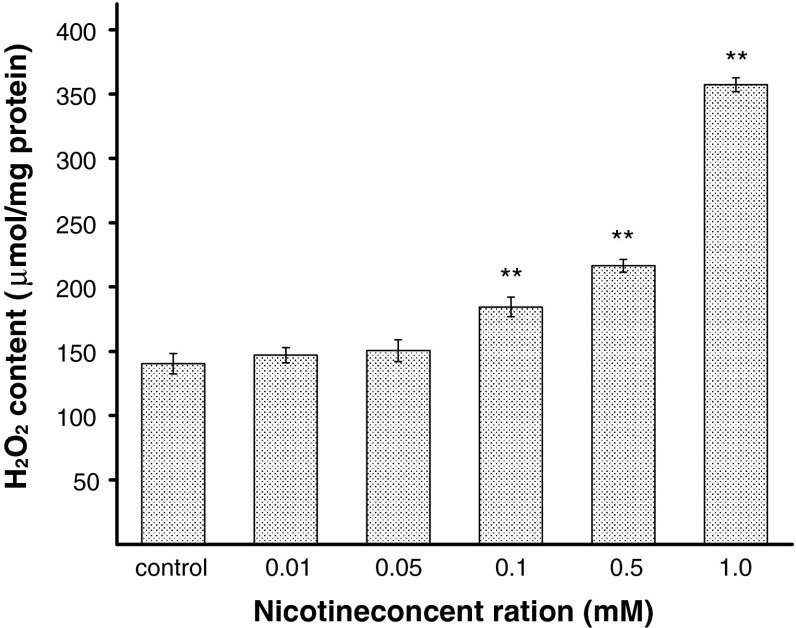
The hydrogen peroxide (H_2_O_2_) content in HEMn-DP cell after 24 h incubation with 0.01, 0.05, 0.1, 0.5, or 1.0 mM of nicotine. Data are mean ± SEM of at least three independent experiments (*n* = 3) performed in triplicate. **p < 0.005 versus the control samples

To explain the effect of nicotine on antioxidant status in melanocytes, the activities of SOD, CAT, and GPx were estimated. The H_2_O_2_ content was also measured. Human melanocytes HEMn-DP were exposed to nicotine in concentrations of 0.01, 0.05, 0.1, 0.5, or 1.0 mM for 24 h. It has been demonstrated that the activity of SOD, i.e., the enzyme which catalyzes the formation of hydrogen peroxide from superoxide anion, increases with increasing concentration of nicotine (Fig. 4). The treatment of cells with 0.05, 0.1, 0.5, or 1.0 mM of nicotine, increased the SOD activity by 9, 24, 32, or 52 %, respectively, as compared with the controls. Nicotine in the concentration of 0.01 mM had no effect on the cellular SOD activity. CAT and GPx work together to catalyze the breakdown of hydrogen peroxide, produced by SOD, to water. The intracellular CAT activity was significantly increased by 26 or 47 % for cells treated with nicotine in concentration of 0.1 or 0.5 mM, respectively, and it increased more than twofold (to 222 %) for cells exposed to nicotine in concentration of 1.0 mM (Fig. 5). In contrast to SOD and CAT, the activity of GPx decreased by 24, 32, and 52 % for cells treated with nicotine in concentrations of 0.1, 0.5, and 1.0 mM, respectively, in comparison to the control cells (Fig. 6). Nicotine enhanced H_2_O_2_ content in melanocytes (Fig. 7). Treatment of cells with 0.1, 0.5, and 1.0 mM of nicotine increased the H_2_O_2_ content by 31, 54, and 154 %, respectively, as compared with the controls. Nicotine in the concentration of 0.01 and 0.05 mM had no effect on H_2_O_2_ content as well as on cellular CAT and GPx activity.

## Discussion

The ability of melanin to interact with many drugs and other chemical substances has both beneficial and adverse effects on the body. Binding of potentially dangerous compounds to this biopolymer protects cells from exposure to excessive concentrations of harmful substances through previous accumulation and further elimination in non-toxic concentrations. However, a long-term exposition to xenobiotics with high affinity for melanin may lead to degeneration of pigmented cells. It is believed that the process of drug-induced damages of melanin-containing tissues takes place when the detoxifying capacities of melanin are exhausted [8, 32]. Nicotine forms complexes with melanin and the amounts of nicotine bound to melanin increase with rising initial drug concentrations and prolongation of incubation time. These complexes were characterized by two classes of independent binding sites with the association constants K_1_ = 2.44 × 10^4^ M^−1^ and K_2_ = 7.72 × 10^2^ M^−1^. The total number of binding sites was estimated to be 1,748 μmol nicotine/mg melanin [33]. It has been demonstrated that the laboratory synthesis of melanin, involving oxidation of tyrosine under the influence of tyrosinase in the presence of ^3^H-nicotine, results in a polymer with incorporated radionuclide [19]. Data from the literature indicate that concentration of nicotine in some organs is related to the pigmentation [14]. Deposition of nicotine in hair containing melanin takes place both during development of hair and after this process [18, 21]. Melanin-containing tissues can store nicotine up to 30 days after a single injection, what was observed in mice [22, 34]. Interactions of nicotine with melanin are still discussed in the context of possible role of the accumulated nicotine in melanin rich tissues, according to nicotine dependence as a topic of health risk significance. That would surely be important, because melanin could act as a non indifferent factor in absorption of nicotine from cigarette smoke and NRT.

In the present study, the effect of nicotine on melanocytes viability, as well as on melanization process and antioxidant defense system in pigmented cells was analyzed. We used the culture of normal human melanocytes HEMn-DP as an in vitro experimental model system. We have found that nicotine in concentrations from 0.0001 to 10 mM decreases the cell viability in a dose-dependent manner (Fig. 1). The value of EC_50_ was determined to be 2.52 mM.

The obtained results show that nicotine affects the melanization process in tested cell line. For concentrations of nicotine 0.01 and 0.05 mM, we observed an activation of melanogenesis expressed by increase in melanin content and tyrosinase activity (Figs. 2, 3). The specific increases in the melanin content for the concentration of nicotine 0.01 and 0.05 mM are probably due to induction of tyrosinase activity by nicotine in these concentrations. The multistep process of melanogenesis is strongly influenced by the key enzyme, tyrosinase. In our study, tyrosinase has increased which correlates with the increasing melanin content. Taking into account the possibility of melanin dispersion occurrence in smokers and in people exposed directly to the effects of nicotine, it can be assumed that the existence of skin coloration may be, among other things, related to the dispersion of melanin-filled melanosomes to the dendrites. It has been shown that smokers’ melanocytes contain melanosomes found in the third or fourth stage of development, entirely filled with melanin, while among non-smokers, melanosomes in the second stage prevail [16]. Nicotine would also activate melanization of melanocytes in an indirect way. It happens through beta-adrenergic effects of epinephrine, which release from the adrenal medulla is augmented while smoking a cigarette. It may cause an increase in the level of cyclic adenosine monophosphate (cAMP), which is an important factor stimulating the biosynthesis of melanin [35]. In humans smoking cigarettes, levels of cAMP in plasma and urine are increased [36]. There might be also a possibility that the high content of melanin can lead to higher absorption of nicotine and lower tobacco cessation rates [14, 23]. A positive correlation between the degree of melanization and the number of cigarettes smoked per day, the level of cotinine—the main metabolite of nicotine in the urine, and test results on the degree of nicotine dependence based on a questionnaire (Fagerström test) was found [23, 37].

For the concentrations from 0.0001 to 0.005 mM as well as for 0.1 mM and 0.5 mM, we observed that the melanin content and tyrosinase activity were similar to control. The lack of increase in the melanin content for the concentrations of nicotine 0.1 and 0.5 mM may be caused by emerging induction of oxidative stress by nicotine that presumably affects the function of tyrosinase enzyme. High H_2_O_2_ content stated for these concentrations may also have inhibitory effect on melanogenic enzymes. For the highest tested concentration of nicotine (1.0 mM) inhibition of melanization process, expressed by reduction of tyrosinase activity and melanin content in melanocytes by about 14 and 16 %, respectively, was demonstrated. This indicates that an inhibitory effect of nicotine in concentration of 1.0 mM on melanogenesis is probably due to its direct inhibition of tyrosinase activity.

In the present study, it has been observed for the first time that nicotine causes significant alterations in the activities of antioxidant enzymes: SOD, CAT, and GPx in melanocytes. The concentration-dependent increase in SOD activity, after exposure of melanocytes to nicotine in concentrations from 0.1 to 1.0 mM (Fig. 4), is probably associated with overproduction of the superoxide anion and subsequent formation of H_2_O_2_ (Fig. 7), which leads to the increase in CAT activity (Fig. 5). We observed decreased activities of GPx for these nicotine concentrations (Fig. 6), what may be explained by the redundant H_2_O_2_ level that cannot be eliminated. After treatment of cells with nicotine in lower concentrations (0.01 and 0.05 mM), the activities of antioxidant enzymes, as well as the H_2_O_2_ content were similar to the controls. Increase of oxidative stress in melanocytes can be observed after exposition to nicotine in concentrations higher than 0.1 mM. The overproduction of ROS may cause damages in basic cellular components of cells resulting in dysfunctions or leading to cell death [38]. Harmful effects of oxidative stress should be overcome by three main enzymes: SOD, CAT, and GPx. Additionally, the scavenger properties of melanin may complement the effectiveness of the antioxidant system of melanocytes. CAT is the main enzyme responsible for degradation of hydrogen peroxide in melanocytes [39]. Thus, it may be possible that CAT and melanin act synergistically to protect cells from oxidative stress, while lower activity of GPx.

To summarize, we have demonstrated that nicotine at lower concentrations induces melanogenesis in normal human melanocytes, having no influence on cellular antioxidant status. At higher nicotine concentrations (above 0.1 mM), the occurence of oxidative stress inside melanocytes (expressed by significant changes of antioxidant enzymes activity) was stated.

## Conclusion

The results presented in this work concerning the effect of nicotine on biochemical processes in normal human melanocytes have to be taken into consideration in the assessment of efficacy and safety of NRT, especially in people with high melanin content.
